# Pork Production with Entire Males: Directions for Control of Boar Taint

**DOI:** 10.3390/ani10091665

**Published:** 2020-09-16

**Authors:** E. James Squires, Christine Bone, Jocelyn Cameron

**Affiliations:** Department of Animal Biosciences, University of Guelph, Guelph, ON N1G 2W1, Canada; cbone@uoguelph.ca (C.B.); jcamer12@uoguelph.ca (J.C.)

**Keywords:** boar taint, androstenone, skatole, synthesis, metabolism, castration

## Abstract

**Simple Summary:**

Castration of male piglets has traditionally been carried out to control boar taint, but animal welfare concerns about surgical castration has brought this practice under scrutiny. In addition, castration decreases growth performance and increases the environmental impact of pork production, so alternatives to castration are needed to control boar taint. In this review, we summarize the current knowledge on boar taint metabolism and outline some key areas that require further study. We also describe some opportunities for controlling the boar taint problem and propose that by defining the differences in metabolic processes and the genetic variations that can lead to boar taint in individual pigs, we can design effective custom solutions for boar taint.

**Abstract:**

Boar taint is caused by the accumulation of androstenone and skatole and other indoles in the fat; this is regulated by the balance between synthesis and degradation of these compounds and can be affected by a number of factors, including environment and management practices, sexual maturity, nutrition, and genetics. Boar taint can be controlled by immunocastration, but this practice has not been accepted in some countries. Genetics offers a long-term solution to the boar taint problem via selective breeding or genome editing. A number of short-term strategies to control boar taint have been proposed, but these can have inconsistent effects and there is too much variability between breeds and individuals to implement a blanket solution for boar taint. Therefore, we propose a precision livestock management approach to developing solutions for controlling taint. This involves determining the differences in metabolic processes and the genetic variations that cause boar taint in specific groups of pigs and using this information to design custom treatments based on the cause of boar taint. Genetic, proteomic or metabolomic profiling can then be used to identify and implement effective solutions for boar taint for specific populations of animals.

## 1. Introduction

### 1.1. Why Is Castration an Issue Now?

In past markets, fatter pigs were most desired, so pigs were castrated to increase the fat content of the carcass. Nowadays, consumer demand has shifted, and leaner meat is considered a healthier option, so to produce a leaner carcass, there is increased desire to raise boars (entire males) over barrows (castrates). Not castrating male pigs will improve growth rate (+13%), increase lean yield (+20%), improve feed conversion (+14%), and decrease feed consumption (−9.5%), which will result in reduced production costs. The faster growth rate and improved feed efficiency will also have a positive environmental impact from reduced energy costs and less manure produced. Raising entire males will thus increase the productivity and sustainability of swine production [1]. 

More recently, surgical castration of male piglets has become a welfare concern due to the pain and stress associated with the procedure. There is also increased cost of pain relief and labour, increased risk of infection and increased pre-weaning mortality due to surgical castration. This has led pig stakeholders in several EU countries to propose a ban on surgical castration (www.boars2018.com), which has now been extended for a few more years due to a lack of suitable alternatives. Eliminating surgical castration would, therefore, have dramatic benefits for both production and consumer acceptance of pork products. The main reason that male piglets are now castrated is to control boar taint, which is an off-odour and off-flavour in pork products from entire male pigs. However, we may also need to control male aggression if boars are not castrated. The pros and cons on various alternatives to surgical castration without pain relief have been recently reviewed [2].

### 1.2. Raising Entire Males

When raising entire males, the welfare issue of castration is removed; however, a new welfare issue arises with increased aggressive and sexual behaviours. Higher levels of aggression have been reported for entire males compared to castrated male pigs [3] and female pigs [4]. Entire males also perform sexual behaviour, such as mounting other males and females, and this behaviour has been observed to cause leg and feet injuries or lameness in 15% of males and 6% of females [5].

Aggression and increased general activity as seen in entire males can increase stress and many studies have found decreased immune function as a result of aggression received [6]. Meat quality also suffers when pigs are in stressful environments, or when receiving aggression or mounting, leading to increased risk of dry, firm, dark meat in entire males [7]. Pigs receiving aggression show decreased average daily gain, which results in a longer time to market and more resources being spent per pig [8].

The use of entire males in pork production may require changes in management practices to reduce negative aggressive and sexual behaviours. Raising boars in single-sex groups results in high incidences of sexual behaviour and injuries compared to mixed-sex or female pens [5]. Raising sibling groups reduces aggressive behaviour in entire males compared to mixing multiple litters; however, there were higher number of bouts of aggression seen in entire males than in castrates [9]. Farrow-to-finish groups have also been seen to reduce androstenone levels in the fat of entire males, suggesting this is a promising management strategy in rearing entire males [9]. Decreasing stocking density [10] and optimizing pen shape and size [11] may also be effective strategies. Additional information on handling and managing boars is available at https://www.boarsontheway.com.

## 2. Boar Taint

Boar taint is caused by the accumulation of high levels of 16-androstene steroids (primarily androstenone), which are produced by Leydig cells in the testis, and skatole and other indoles, which are produced from the metabolism of tryptophan by the gut microflora (reviewed in [12]). 

Androstenone (5α-androst-16-ene-3-one) is a major component of boar taint produced by the testis at sexual maturity. Its physiological role is a sex pheromone which regulates reproductive development and behaviour in female pigs. Androstenone was first attributed as a cause of boar taint by Patterson [13] and androstenone metabolism has been extensively studied by Gower [14]. More recently the importance of androstenone metabolites, e.g., androstenols, in boar taint has also been considered [15].

Skatole (3-methylindole) is produced by bacterial metabolism of tryptophan in the hindgut. It was not initially recognized as a contributor to boar taint but was eventually identified by Vold [16] and Walstra & Maarse [17] as an important component of boar taint. Skatole production is predominantly influenced by diet, but also by genetics and environmental factors. There is also evidence that indoles and skatole metabolites can contribute to boar taint.

Other potential contributing compounds to boar taint include aldehydes and short-chain fatty acids, phenols (p-cresol, 4-ethylphenol) and 4-phenyl-3-buten-2-one. It may be that these compounds act by enhancing the effects of skatole and androstenone to cause boar taint [12].

The accumulation of these boar taint compounds in fatty tissues is regulated by the balance between synthesis and degradation of these compounds. This can be affected by various factors, including environment and management practices, sexual maturity, nutrition, and genetics. In this review, we summarize the current knowledge on boar taint metabolism and outline some key areas that require further study. We also describe some opportunities for controlling the boar taint problem and propose that by defining the differences in metabolic processes and the genetic variations that can lead to boar taint in individual pigs, we can design effective custom solutions for boar taint.

## 3. Boar Taint Metabolism

### 3.1. Androstenone Synthesis, Metabolism and Transport

**Figure 1:**
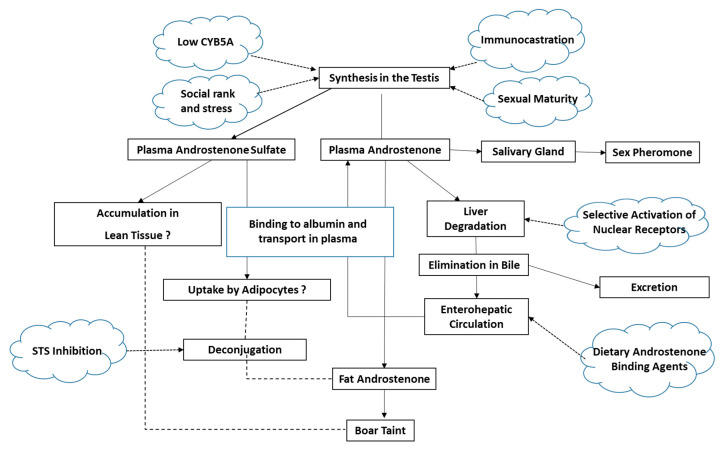
Flow chart of androstenone synthesis, metabolism and transport and factors affecting boar taint.

Androstenone is synthesized in the testis and enters the blood stream to be transported to peripheral tissues where it accumulates to cause boar taint. It is metabolized and eliminated by the liver (Figure 1).

#### 3.1.1. Androstenone Synthesis 

**Figure 2:**
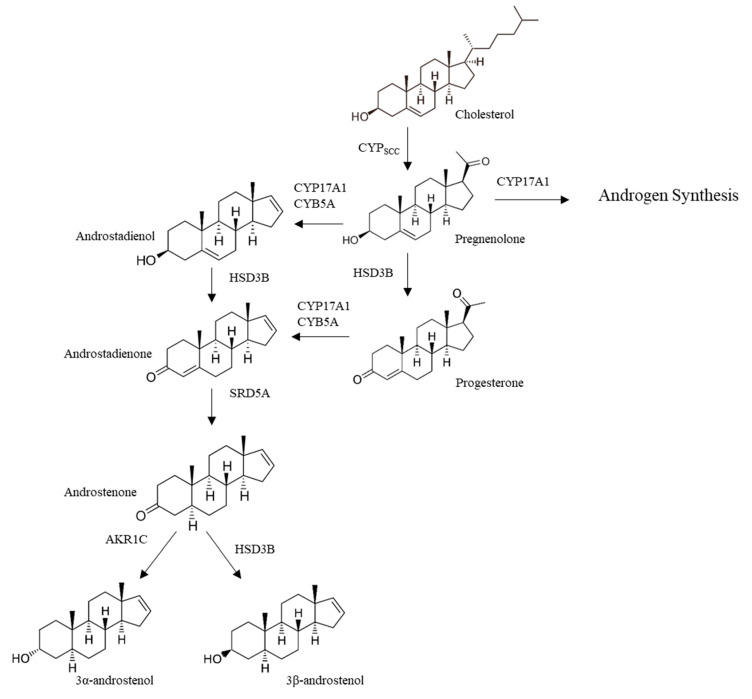
Pathways of androstenone synthesis and metabolism.

The first step in the synthesis of 16-androstene steroids is the formation of 5,16-androstadien-3β-ol (androstadienol) from pregnenolone [14]. Androstadienol is then converted to 4,16-androstadien-3-one (androstadienone), which is further metabolized by steroid 5α-reductase (SRD5A) to produce androstenone (5α-androst-16-ene-3-one) [18,19]. Two isoforms of this enzyme exist in the pig and it has been shown that it is the SRD5A1 rather than the SRD5A2 isoform that is expressed in the testis and is correlated with levels of androstenone in fat [20,21]. Androstenone can then be reduced by either 3β-hydroxysteroid dehydrogenase (HSD3B) to produce 3β-androstenol or aldo-keto reductase (AKR1C) to 3α-androstenol [14] (Figure 2). Following synthesis in the testis, the 16-androstene steroids are released through the spermatic vein into the circulation and accumulate in fatty tissue to cause boar taint. They are also transported to the submaxillary salivary gland, where they bind to the lipocalin protein pheromaxein, and are released in the saliva as sex pheromones to regulate female reproduction [22].

The synthesis of androstadienol from pregnenolone is catalyzed by the andien-β synthase system [14] which includes cytochrome P45017A1 (CYP17A1), with cytochrome b5 (CYB5A) acting as an electron shuttle with cytochrome P450 reductase (POR). CYP17A1 also catalyzes the 17,20-lyase reaction responsible for the synthesis the androgen dehydroepiandrosterone (DHEA) from pregnenolone; this reaction is also mediated by CYB5A, which interacts allosterically with the CYP17A1-POR complex [23]. This step is thus a key branch point where the synthesis of the 16-androstene steroids and androgens diverge; when levels of CYB5A are limiting, the production of androstadienol is significantly reduced [24]. A recent study [25] identified amino acid residues in CYB5A and CYP17A1 that affected the relative production of androstadienol and DHEA from pregnenolone. Introducing mutations of these amino acids into pigs may be an effective method to decrease androstenone synthesis while maintaining sex steroid synthesis in the testis. 

#### 3.1.2. Androstenone Metabolism 

Androstenone is metabolized in a two-phase process that occurs primarily in the testes and liver. Phase I metabolism produces androstenol metabolites of androstenone through 3α-hydroxy and 3β-hydroxy reduction [26]. Androstenone and its metabolites can then be conjugated to a sulfate or glucuronide group during Phase II metabolism by the cytosolic sulfotransferase enzyme SULT2A1, or by UDP-glucuronosyltransferase (UGTs), respectively [27,28]. It was suggested from Western blotting analysis that SULT2B1 may also be involved in the sulfoconjugation of androstenone [29], but subsequent cloning and expression of porcine SULT2B1 demonstrated that this was not the case [27].

**Figure 3:**
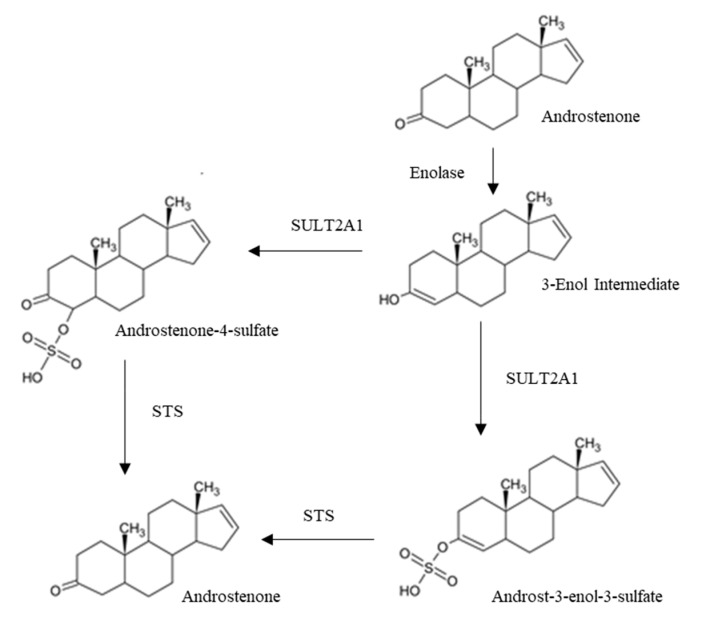
Propose mechanism for the reversible sulfation of androstenone.

Approximately 70% of the 16-androstene steroids produced in the Leydig cells are present as sulfoconjugates in the peripheral circulation [30,31]; no glucuronidated forms of androstenone have been identified from Leydig cell culture. Leydig cells produce two forms of androstenone sulfate, tentatively identified as androst-3-enol-3-sulfate and androstenone-4-sulfate, which both regenerate the parent compound androstenone and not a hydroxylated metabolite following removal of the sulfate group [31] (Figure 3). This suggests that androstenone sulfate may function like many steroid sulfates, acting as a steroid reservoir that can be enzymatically deconjugated by steroid sulfatase (STS) to return free androstenone [31,32]. 

Androstenone sulfate may potentially also accumulate in hydrophilic lean tissue and contribute to boar taint. Ampuero Kragten et al. [33] found that levels of free androstenone in muscle and intramuscular fat are not well correlated. Androstenone is normally measured in fat, while sensory analysis of boar taint typically uses both fat and muscle, so the presence of androstenone sulfate in muscle may explain discrepancies between sensory scores and fat androstenone concentrations that were first noted by Bonneau et al. [34]. However, the possibility that androstenone sulfate can accumulate in muscle tissue and contribute to boar taint has not yet been investigated.

Androstenone metabolism also occurs in the liver. Human liver expresses the sulfotransferase enzymes SULT1A1, SULT1B1 and SULT2A1 [35] and SULT2A1 and SULT2B1 are expressed in porcine liver [20]; these enzymes facilitate hepatic sulfoconjugation. Sulfated steroids are then incorporated into the bile and secreted into the gut, where they can undergo subsequent deconjugation by the gut microflora [35,36]. Steroids can then be reabsorbed across the intestinal wall, which enables the recycling of these steroids in a process known as the enterohepatic circulation [36]. We have proposed interrupting the enterohepatic circulation as a potential approach to decreasing boar taint from androstenone [37].

Robic et al. [20] have reported on the expression of genes involved in the metabolism of steroids in testis, adrenal gland, adipose tissue and liver. They concluded that porcine adrenal glands produce corticosteroids almost exclusively and that biosynthesis of steroids does not occur in adipose tissue.

#### 3.1.3. Androstenone Transport

Lipophilic steroids normally travel in the circulation bound to carrier proteins, which renders them inactive. According to the “free hormone hypothesis”, biologically active steroids are released from carrier proteins and subsequently enter target tissues by diffusion [38]. The sex hormone binding globulin is a carrier protein responsible for the transport of sex steroids such as testosterone, estrone and DHEA in the plasma; however, it is absent in the porcine plasma proteome [39,40]. Zamaratskaia et al. [40] used a competitive binding assay to study binding of androstenone to plasma proteins. This group did not find a specific binding protein for androstenone in the plasma and suggested that androstenone likely binds to albumin or alpha-globulins due to their non-specific binding properties and abundance in porcine plasma. Subsequent work from our laboratory using a novel high-performance liquid chromatography (HPLC) gel filtration technique [41] confirmed that androstenone is bound and transported in the plasma of the boar by albumin. Approximately 80% of androstenone in the plasma is bound to albumin, which dissociates to drive the accumulation of androstenone in the adipose tissue [41]. However, the percentage of androstenone displaced from albumin can vary considerably between different animals. In a subsequent study, it was found that the binding of androstenone to albumin was lower in animals that had high concentrations of androstenone in the fat than in animals with low fat androstenone concentrations [42]. These results suggest that the amount of binding of androstenone in the plasma could affect the accumulation of androstenone in fat and thus function as an important biomarker for identification of animals with a high potential for boar taint. 

#### 3.1.4. Aspects That Are Not Yet Well Understood

Squires et al. [25] have identified amino acid residues in CYB5A and CYP17A1 that affect the relative production of androstadienol and DHEA from pregnenolone. A more complete understanding of the interaction between CYB5A and CYP17A1 could lead to methods for the specific inhibition of androstenone synthesis; for example, small-molecule inhibitors could be designed to alter this interaction, or gene editing could be used to alter the sequence of CYB5A and CYP17A1 (see Section 4.6.2).

The role of the enterohepatic circulation in regulating androstenone levels is not completely understood. Proof-of-concept studies have shown that adding activated charcoal to finishing diets can decrease androstenone levels [37], but the mechanism of the enterohepatic circulation of androstenone has not been described. There is also a need to find cost effective and selective adsorbents for androstenone that can be added to finishing diets of entire males.

The mechanism of accumulation of androstenone and potentially androstenone sulfate by adipose tissue and other tissues of the boar has yet to be investigated. In humans, the uptake and subsequent deconjugation of steroid sulfates occur in various tissues, which have STS activity and express specific membrane transporters belonging to the solute carrier organic anion transporting polypeptide (OATP) family [43,44]. The membrane transporters OATP-B (OATP2B1), OATP-D (OATP3A1) and OATP-E (OATP4A1), which facilitate the Na+ dependent uptake of DHEAS into the placenta, mammary gland and temporal lobe, are constitutively expressed in the adipose tissue of humans along with STS [44,45]. If this is also the case for pigs, the development of boar taint may also be regulated by the rate of accumulation of androstenone or androstenone sulfate in target tissues as well as the balance between its synthesis and metabolism [12]. Consequently, animals expressing high levels of SULT2A1 in the testes and liver as well as high levels of STS in important peripheral tissues such as the fat may have a high potential for developing boar taint resulting from the indirect accumulation of androstenone from androstenone sulfate. For this reason, the expression of SULT2A1 and STS may be valuable biomarkers for boar taint.

Future studies investigating the role of androstenone sulfate in the boar should examine the binding characteristics of androstenone sulfate in the plasma as well as the ability of androstenone sulfate to accumulate directly or indirectly in peripheral tissues. Important questions to be considered for these studies include: What is the binding affinity of androstenone sulfate for transport proteins in porcine plasma? Does the binding of androstenone sulfate in the plasma affect the development of boar taint? Can androstenone sulfate accumulate in the lean or fat tissue and how? These studies should focus on comparing the transport and potential uptake of androstenone sulfate between animals confirmed to have either high or low concentrations of androstenone in the fat to identify further sources of inter-animal variability that contribute to the development of boar taint.

### 3.2. Skatole Synthesis and Metabolism

**Figure 4:**
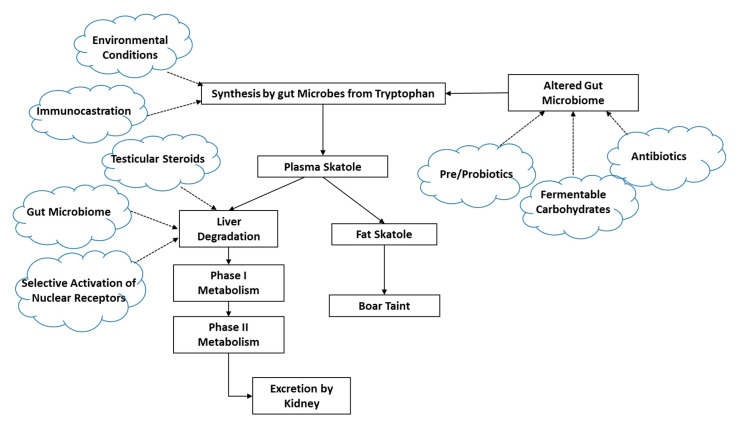
Flow chart of skatole synthesis and metabolism and factors affecting boar taint.

Skatole is produced by the bacterial breakdown of tryptophan in the hind gut, and skatole production is affected by diet as well as the composition of the gut microflora. Skatole metabolism and clearance is regulated by the activity of the Phase I and Phase II enzymes in the liver that degrade skatole. The balance between production and clearance of skatole determines how much skatole accumulates in fat (Figure 4). The physiological mechanisms and nutritional effects on skatole synthesis and metabolism leading to high skatole levels in fat have been reviewed [46].

#### 3.2.1. Skatole Production 

The synthesis of skatole in the gut can be affected by: 1. the availability of tryptophan from either low digestible feed or cell debris; 2. the presence of a microbiome composition that is appropriate for skatole synthesis; and 3. conditions which promote the bacterial metabolism of tryptophan to skatole, rather than for the synthesis of bacterial protein, such as when insufficient carbohydrates are available for energy production [46]. The amount of skatole absorbed into the blood is proportional to the amount produced in the hindgut [47,48] and high levels of skatole in peripheral blood promote skatole accumulation in adipose tissue.

There are differences in skatole production among individual pigs that are fed the same diet [49] and levels of skatole in feces can be used to identify animals that produce high levels of skatole [49,50]. This is an important consideration for studying skatole metabolism in vivo, since pigs with high levels of skatole production are needed for these studies. For example, Lanthier et al. [49] found that in entire male pigs at 42 days of age, hepatic SULT1A1 activity was negatively correlated with skatole levels in plasma and fat, and this correlation was improved when the concentration of skatole in cecal contents was included in the regression analysis.

The gut of the newborn is rapidly colonized by bacteria from the maternal environment and a stable, complex and diverse bacterial community develops after weaning. This process is affected by diet, environment and host genetic factors. Many studies have demonstrated that the composition of the gut microbiota can affect immunological and metabolic pathways of the host and thereby affect the health and performance of domestic animals [51]. This is thought to be mediated through the production of metabolites by the gut microbiota, such as short-chain fatty acids from fermentation of dietary fibre and metabolites from amino acid metabolism, including skatole and other indoles. There is also tremendous temporal variation in the composition of the gut microbiota within individuals and between individuals, and this may in part account for the individual differences in skatole production. Skatole production has been confirmed in four bacterial species of two genera, *Clostridium* and *Olsenella* [50]. 

Skatole production can be reduced by including sources of fermentable carbohydrates (e.g., inulin, fructooligosaccharides, raw potato starch, sugar beet pulp, chicory inulin, or high amylase barley) in the diet. Three hypotheses have been proposed to explain the effects of fermentable fibre: 1. fermentation produces butyrate, which reduces cell apoptosis and decreases the amount of cellular debris that is a source of tryptophan for skatole production; 2. fermentable fibre increases overall microbial activity and incorporation of available tryptophan into bacterial biomass, which decreases the amount of tryptophan available for skatole production; and 3. the composition of the microbiota is altered to decrease the amount of skatole-producing bacteria. A recent study [50] demonstrated that butyrate treatment does not decrease skatole production and feeding diets supplemented with chicory does not decrease the population of skatole-producing bacteria. This suggests that the mode of action of feeding chicory roots is likely through increasing bacterial biomass and thus decreasing the amount of tryptophan available for skatole production. However, feeding chicory root can also result in changes in skatole metabolism by increased hepatic expression of *CYP2E1*, *CYP2A* and *CYP3A* in pigs; secondary metabolites of chicory induced these CYPs in porcine hepatocytes (reviewed in [52]).

Various antibiotics can also affect skatole production by altering the composition of the gut microflora (reviewed in [12]). The antibiotic activity of selected plant extracts (e.g., garlic oil) may also be effective in altering the gut microflora [46]. Skatole can also be absorbed from the manure, so dirty pigs of either sex can accumulate skatole in fat, especially in warmer temperatures [53]. Earlier studies demonstrated that skatole production is decreased by feeding bicarbonate; the increased pH promotes the synthesis of indole rather than skatole [54].

A recent study [55] examined how immunocastration (immunization against GnRH) decreases the production of skatole in the gut. Immunocastration decreased levels of IGF1, which is proposed to decrease turnover of mucosal cells in the ileum and colon [56]. Levels of testicular steroids were also decreased by immunocastration, leading to increased expression of key genes involved in skatole metabolism in the liver (*CYP2E1*, *CYP2A*, *CYP2C49* and *CYB5A*), which will enhance skatole degradation and clearance.

#### 3.2.2. Skatole Metabolism and Clearance 

**Figure 5:**
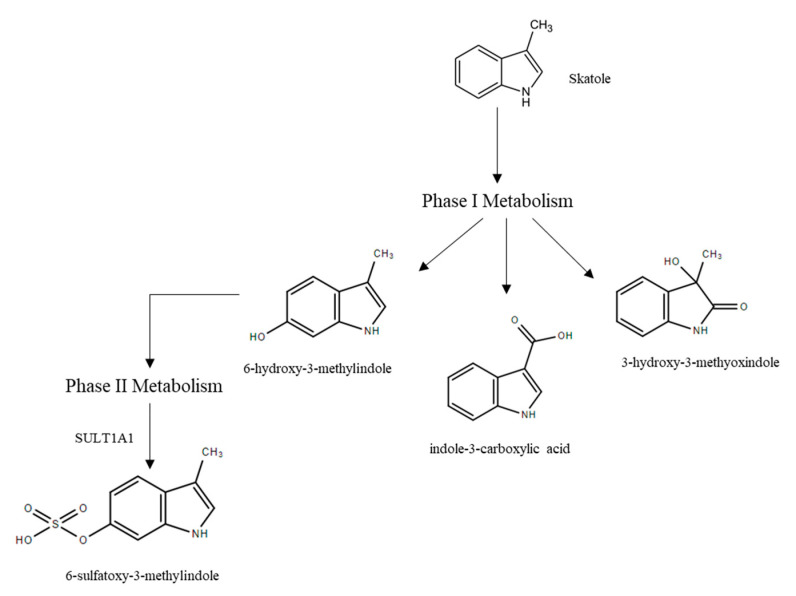
Pathways of skatole metabolism.

Skatole metabolism occurs primarily in the liver and involves two phases; Phase I involves the addition of a hydroxyl group that can be used to attach a conjugate in Phase II (Figure 5). The majority of Phase I metabolism occurs through the cytochrome P450 (CYP450) system, which is a superfamily of heme-containing mono-oxygenase enzymes located in the endoplasmic reticulum. Seven Phase I metabolites of skatole have been identified [57] and several of these metabolites undergo subsequent Phase II sulfoconjugation reactions via SULT1A1 [58]. Production of high levels of the metabolite 6-sulfatoxy-3-methylindole (6-OH-3MI sulfate) has been suggested as a biomarker of entire male pigs that are able to efficiently metabolize and excrete skatole, while high levels of 3-hydroxy-3-methyoxindole (HMOI) are associated with decreased clearance of skatole [59]. Levels of skatole metabolites HMOI and indole-3-carboxylic acid in urine and HMOI in plasma have been suggested as biomarkers of high skatole levels in fat [60], since levels of these metabolites were correlated with skatole levels in fat. 

Several different animal studies have identified the cytochrome P450 isoforms CYP2E1 and CYP2A19 as the main metabolizers of skatole (reviewed in [12]). These studies used indicator substrates, inhibitors or antibodies to assess the activities of the different CYP450 isoforms, but in most cases these reagents were not validated for their specificity in pigs. We have investigated this question by expressing six different individual porcine CYP450 isoforms along with CYB5A in HEK cells and measuring their role in the metabolism of skatole [61]. This in vitro cell culture system eliminates the problems of lack of specificity of antibodies, inhibitors and substrates for CYP450 isoforms in the pig and the contributions of any other CYP450s that would be present in tissue samples. We found that pig CYP1A1, CYP2A19, CYP2C33v4, CYP2C49, CYP2E1 and CYP3A and human CYP2E1 were all capable of producing skatole metabolites. CYP2A19 produced the highest amount of the key metabolite 6-hydroxy-3-methylindole (6-OH-3MI), followed by porcine CYP2E1 and CYP2C49. Co-transfection with CYB5A increased the production of skatole metabolites by some of the CYP450s, suggesting that CYB5A plays an important role in the metabolism of skatole.

Skatole is produced in the gut of both males and female pigs; however, the production of sex steroids at the onset of puberty in males is thought to inhibit the hepatic metabolism and clearance of skatole, which increases the accumulation of skatole in entire males [12]. The addition of physiological concentrations of androstenone reduced the expression of CYP2E1 and CYP2A in pig hepatocytes and activity in liver microsomes [62,63]. Some dietary factors such as chicory roots can also affect activity of CYP450 [52] and alter the expression of skatole-metabolizing enzymes. Other natural products (e.g., phytoestrogens) which are ligands for the nuclear receptors constitutive androstane receptor (CAR), pregnane X receptor (PXR) and farnesoid X receptor (FXR) can also regulate skatole metabolism.

#### 3.2.3. Aspects That Are Not Yet Well Understood

Future studies on skatole synthesis and metabolism should include investigating the role of the gut microbiome and the genetics of the individual pig on the production and absorption of skatole from the gut and metabolism by the liver. Important questions include: What affects the availability of tryptophan for skatole synthesis? What is the ideal microbiome for reduced skatole production? How does the genetics of the pig affect the microbiome? How does the microbiome regulate skatole metabolism and clearance in the pig? 

For studies on skatole metabolism, the concentration of skatole in feces should be measured to identify those animals that are high producers of skatole. The enumeration of the amounts of different bacteria in feces can be accomplished by real-time PCR of bacterial genomic DNA using species specific primers. Differences in the composition of the microbial communities in the gut can be assessed based on metagenomics sequencing of regions of the 16S rRNA gene [51]. This data can be compared to levels of skatole in the feces and backfat, genotypes of key genetic markers and expression of key boar taint genes, and the hepatic production of skatole metabolites.

A practical experimental method for establishing pigs with a homogenous microbial profile in the hind gut has recently been developed [64]. This involves raising newborn piglets in high efficiency particulate air (HEPA) filtered isolators and feeding them artificial colostrum containing fecal material from the same group of sows. This may be a good tool for studies investigating the role of the gut microbiota in skatole production. 

We have shown that nuclear receptors CAR, PXR and FXR can affect the metabolism of boar taint compounds [65,66]. In Leydig cells, activation of CAR, PXR, or FXR increased the expression of several genes involved in steroidogenesis, including *CYB5A* and cytochrome B5 reductase 1 (*CYB5R1*), as well as hydroxysteroid (17-beta) dehydrogenase 4 (*HSD17B4*) and retinol dehydrogenase 12 (*RDH12*). Activation of CAR or PXR decreased sex steroid production and increased production of 16-androstene steroids, while activation of FXR decreased sex steroid production. Thus, activation of these nuclear receptors may lead to increased levels of 16-androstene steroids, likely by altering the activity of CYP17A1 through CYB5A and CYB5R1. In hepatocytes, activation of CAR, PXR, or FXR increased expression of *CYP2B22* by CAR, of *CYP2A19*, *CYP2B22*, *CYP2C33*, and *CYP2C49* by PXR, of *CYP2C33* and *CYP2E1* by FXR, and of *CYP19A2* by all three receptors. Activation of PXR dramatically increased the amount of androstenone metabolism. FXR activation increased the expression of *CYP2E1* and the production of the key metabolite 6-OH-3MI. This suggests that selective activation of PXR and FXR may decrease boar taint by increasing the hepatic metabolism of androstenone and skatole, but this needs to be confirmed using whole animal studies.

## 4. Potential Solutions for the Boar Taint Problem

### 4.1. Assessment of Boar Taint and Carcass Sorting

One potential approach for dealing with boar taint is to measure the amount of boar taint in a carcass and then sort out those carcasses that have high boar taint and are not suitable for fresh pork products. This tainted meat can be used in various types of processed products where the boar taint is masked by adding spices, curing or smoking, preferably in processed products that are served cold (reviewed in [67]). The meat can also be mixed with untainted meat to dilute the amount of boar taint. In order to use this carcass sorting strategy, there must be a low percentage of tainted carcasses, since these have a significantly lower value. Using specific low-taint breeds or lighter-weight carcasses (see below) could help achieve this requirement. 

Measurement of taint and sorting carcasses is also technically challenging for slaughter plants. The main limitation is the lack of rapid online assessment tool(s) for boar taint. Several types of sensors have been developed (e.g., electronic nose and biosensors), but these have not yet been developed into commercially useful products. A number of rapid analytical methods (e.g., chromatography, mass spectrometry, and immunoassays) are available, but these are more suitable for offline laboratory-based analysis [68]. There will also be logistical problems to accommodate space for testing and sorting carcasses on the slaughter line. The human nose scoring sensory test has been adopted in some countries to detect boar taint in commercial plants [69].

### 4.2. Dietary Approaches

Several different dietary approaches have been proposed to reduce boar taint, especially for skatole, and these have been extensively reviewed [46]. Skatole levels can be decreased by including fermentable carbohydrates in the diet that can alter the activity and composition of the gut microflora, to decrease the formation of skatole in the hindgut. There are feed products on the market that combine fibres and feed additives to reduce skatole levels. These are available from Dumoulin in Belgium and Vitelia in the Netherlands and are given to boars in the last few weeks before slaughter [70]. There is also potential for use of other prebiotic and probiotic compounds to manipulate the microbiome. This may also improve skatole metabolism in the liver by increasing the expression and activity of key enzymes involved in skatole metabolism, as well as having beneficial effects on growth performance.

In contrast to skatole, there have been only limited nutritional approaches to decrease androstenone levels. Feeding diets to maximize growth rate so that entire males reach market weight before they become sexually mature may help to reduce androstenone levels. However, the time of puberty is affected by the breed and also varies between individuals within a breed, so this is not always effective in eliminating boar taint (reviewed in [12]). Furthermore, a reduction in slaughter weight reduces the amount of meat yield from a carcass and is thus not economically attractive.

Androstenone is metabolized in the liver and the metabolites move into the digestive tract via the bile. They can then undergo subsequent deconjugation by the gut microflora and be reabsorbed back into the body via the enterohepatic circulation. We have proposed decreasing androstenone levels by adding non-nutritive sorbent materials to finishing diets, which can bind to androstenone in the gut and prevent its reuptake [37]. In a pilot study, activated charcoal supplemented in the feed effectively decreased fat androstenone concentrations to below threshold values for boar taint [37]. This is a promising approach, but further detailed studies are needed to determine the mechanism and assess the efficacy of this technology under commercial production conditions. There is also a need to identify androstenone-specific, low cost binding adsorbents that are effective alternatives to activated charcoal, and to determine their effective dose and treatment time for specific groups of pigs. 

There is also the potential to use various natural products to activate nuclear receptors (reviewed in [71]) and affect hepatic metabolism of both boar taint compounds and synthesis of androstenone in Leydig cells [65,66]. In Leydig cells, activation of CAR or PXR decreased sex steroid production and increased production of androstenone, while activation of FXR decreased sex steroid production [65]. In hepatocytes, activation of CAR, PXR, or FXR increased metabolism of skatole, while activation of PXR dramatically increased androstenone metabolism [66]. This suggests the potential use of natural products to activate nuclear receptors regulating boar taint metabolism. Natural products that can activate nuclear receptors include diallyl sulfide, an active ingredient of garlic, a CAR agonist; (Z)-gugglesterone from *Commiphora mukul,* an inverse agonist of CAR and agonist of PXR; hyperforin, from St. John’s Wort (*Hypericum perforatum*), a modulator of PXR; coumestrol, a phytoestrogen that acts as a PXR antagonist; oleanolic acid, which is common to traditional Chinese herbal medicines, which is a selective modulator of FXR.

### 4.3. Environmental and Management Approaches

Skatole levels can be affected by the housing environment and management. Keeping pigs in pens soiled with feces and urine in high stocking rates significantly increased indole and skatole levels in backfat compared to clean pigs, especially during higher summer temperatures [53]. Under these conditions, gilts can also have high levels of skatole in fat, so maintaining a clean environment helps to decrease boar taint from skatole. Shorter daylight accelerates the onset of puberty; thus, boars reared in autumn/winter have increased plasma levels of testosterone and estradiol, fat skatole and testis weights. However, there was no clear seasonal effect on fat androstenone [72]. 

Androstenone (as well as testosterone) levels are higher in more dominant and higher-ranking males within a group [73]. Pre-slaughter handling and transport has been reported to increase levels of androstenone, skatole and indole in fat [74]. Further work is needed to better understand how these factors can be best managed to control boar taint.

Aggression in entire males is generally attributed to androgens, including testosterone, that are produced in the testes; thus, these behaviours are alleviated by removal of the testes [75]. There is a lot of evidence supporting the connection of androgens and aggression, but there have been few studies investigating the effect of androstenone or boar taint on aggression in boars. Androstenone has been correlated with dominance ranking of boars in same-sex pens, and higher androstenone levels of the dominant boar also showed stimulating effects, increasing androstenone concentrations in the subordinate boars of the same pen [73]. However, another study [76] showed no association between aggression and androstenone in entire male pigs. There appears to be no clear link between levels of androstenone or boar taint and aggressive or sexual behaviours in current literature as there is with androgens.

The impact of handling of pigs before and during transportation to the slaughterhouse on poor welfare, stress and meat quality has been investigated [77,78,79], but the effect on boar taint outcomes has not been extensively studied. Wesoly et al. [74] found that transportation time is positively correlated with androstenone concentrations, indicating that longer transport from farm to abattoir can increase androstenone levels. In a pilot study from our group (Cameron et al., unpublished), we found a significant increase in plasma androstenone levels immediately after transportation and slaughter compared to plasma androstenone levels 4 days prior to slaughter. Testosterone concentrations increase with sexual stimuli or hCG stimulation [80] and changes in plasma androstenone may also occur in response to stressors such as transport and fighting. Reducing transport and lairage time may therefore be a method to reduce androstenone concentrations. Further research should investigate the extent of androstenone increase in adipose tissue from pre-slaughter to slaughter conditions with various transport times and handling regimens.

### 4.4. Effect of Sexual Maturity 

Both androstenone and skatole levels vary with age [81]; it is therefore necessary to consider sexual maturity when assessing the potential for boar taint, so that boars are old enough to have a mature, functional testis and can express their genetic potential for boar taint. Three possible boar taint phenotypes for androstenone have been described [12]. Boars can be early maturing, with a high potential for androstenone production, late maturing with a high potential for androstenone production, or have a low potential for androstenone production even when sexually mature. In order to distinguish between these latter two groups, it is necessary to assess the degree of sexual maturity. This can be done by measuring plasma levels of sex steroids such as estrogens or the ratio between testis weight and body weight [72].

Early postnatal pigs have a peak in plasma levels of steroid hormones at 3–4 weeks of age [82] and this early rise in steroidogenesis influences the animal’s later development and behaviour [83]. There is also an increase in plasma levels of androstenone at 3–4 weeks of age [84]. Blocking this early increase in steroidogenesis did not affect the subsequent increase in androstenone production and boar taint at puberty [84], but we have proposed that the amount of steroid production in early life can be used to predict the amount of steroid production at maturity. Our preliminary results suggest that plasma androstenone concentrations at 21 days of age may be indicative of the extent of androstenone accumulation in the fat and plasma in pigs slaughtered at over 120 kg live weight. This can aid with identifying boars at risk of developing boar taint early, so that solutions such as immunocastration can be implemented [85]. 

### 4.5. Immunocastration

Boar taint can be reliably controlled by immunocastration using a commercially available vaccine (Improvest^®^ from Zoetis) which stimulates the production of antibodies against gonadotropin releasing hormone (GnRH). GnRH is produced by the hypothalamus in the brain to drive the release of luteinizing hormone by the pituitary gland, which stimulates steroid hormone synthesis and the development of the testis. The vaccine induces the production of antibodies that inactivate GnRH to shut down testicular development to the same extent as surgical castration and eliminate the production of both androstenone and the sex steroids. Levels of skatole are also low in immunocastrated pigs, and this is most likely due to enhanced metabolic clearance by the liver after steroid production is suppressed, as occurs in surgically castrated pigs [55]. A meta-analysis of Batorek et al. [86] has summarized data on the effectiveness of immunocastration for reducing levels of both androstenone and skatole. The procedure is non-invasive and lowers the risk of infection and pain from surgical castration.

The growth rate, feed conversion and lean yield of immunocastrates are closer to that of entire males than to barrows. The immunization procedure involves injection of an initial priming dose at 8–9 weeks of age followed by a second activating dose at 4–6 weeks before slaughter. Males receiving the first vaccination continue to grow like entire males, but after the second vaccination the immunocastrates consume more feed and grow more rapidly than entire males. However, delaying the timing of the second dose from 6 to 4 weeks before slaughter does not dramatically affect production efficiency and meat quality [87]. The initial dose does not produce a large antibody response, so the procedure could potentially be used as part of the selection process for breeding boars, where all boars would be given the first injection and only non-selected boars would get the second dose and are then immunocastrated.

Immunocastration also reduces aggressive and sexual behaviours [88] with head knocking, fighting and mounting behaviours reduced significantly compared to entire males, making the immunocastrated pigs not behaviourally different from barrows [89,90]. Most recent research is focusing on implementing immunocastration into swine production systems [91]. Immunocastration is used extensively in swine operations in Brazil, Australia and New Zealand, but there has not been wide acceptance of the immunocastration technology in North America or in the EU. This is due to lack of acceptance by consumers and also lack of acceptance of males at the slaughterhouse, due to increased labour and equipment modifications needed to handle entire males. There is also the potential for accidental self-injection of workers, but procedures are in place to minimize this problem. 

### 4.6. Role of Genetics 

The genetic effects on boar taint are related to differences in the production and clearance of taint compounds and the degree of sexual maturity at slaughter, as described above. The production of low boar taint lines of pigs using genetic approaches would be a long-term solution for the boar taint problem. Boar taint can potentially be reduced by selective breeding through performance testing or using genetic markers, or by genome editing of genes involved in boar taint.

#### 4.6.1. Selective Breeding

Genetic selection of low boar taint animals offers a non-invasive, cost effective, welfare friendly and potential long-term solution to the issue of boar taint. The amount of boar taint can vary dramatically between different breeds (reviewed in [12]) and there are also differences in boar taint among individual pigs within a breed. Heritability estimates for androstenone and skatole are moderate to high, and there is a genetic correlation between androstenone and skatole [92], so selection for low boar taint is possible. However, producing entire males can also raise other welfare issues, so genetic selection for reduction in androstenone must also be accompanied by control of aggressive and sexual behaviours. 

Genomic selection has been used to reduce boar taint in some countries and several breeding companies have developed ‘low-taint’ products. Topigs Norsvin has developed a method to select low-taint boars. Similarly, Nucleus in France offers Piétrain selected low-taint reproducers, labelled “INO”. A low-taint line has also been developed by the University of Bonn in Germany, in partnership with the breeding company GFS and the slaughterhouse Westfleisch. The retailer Delhaize in Belgium has worked with breeding company PIC to develop a low-taint line for a castration-free pork label [70]. 

Baes et al., [92] have developed a performance test for boar taint using a biopsy device to obtain fat samples from live breeding boars for analysis of boar taint compounds. This allows the direct testing of breeding candidates for the boar taint phenotype, so that boar taint can be measured directly and not estimated from full sibs or progeny testing. The degree of sexual maturity of the boars tested also needs to be assessed so that the boar taint phenotype can be accurately defined [11]. However, the use of performance testing for reducing boar taint is complicated by potential negative correlated effects on fertility [92] and other traits of economic interest. The identification of QTLs or genes that specifically affect only boar taint will allow specific selection methods to reduce boar taint to be developed without negative pleiotropic effects [93] (see the Animal Quantitative Trait Loci (QTL) Database (Animal QTLdb) at https://www.animalgenome.org). The genetic correlations of androstenone with production traits such as growth rate, feed efficiency or carcass quality are mostly favourable, and selection applied in sire lines should tend to decrease boar taint. In dam lines, the genetic relationships of boar taint with reproductive traits differs between breeds, so breeding to reduce boar taint needs to be carefully monitored in order to not adversely affect reproduction [94]. 

The pathways of synthesis and metabolism of androstenone and skatole metabolism have mostly been described (Figure 2 and Figure 5) and the genes involved in these pathways have been identified through metabolism studies [12,19,95]. GWAS studies using large SNP panels for detection of QTL have located several loci throughout the genome [93] and fine mapping of these loci has identified important candidate genes [96]. Several expression studies of candidate genes have also been carried out in both testis and liver using microarrays, and mRNA sequencing has been used to identify differentially expressed genes [97].

Our group has focused on identifying non-synonymous SNPs in candidate genes involved in the metabolism of boar taint compounds [98,99]. We now have a set of 1400 markers in 200 candidate genes and are validating the marker set in Canadian lines of pigs for use in breeding programs to generate pigs that have reduced boar taint [100]. In our work, we assembled a list of candidate genes involved in boar taint, identified SNPs within these genes and then determined if these SNPs are effective genetic markers associated with boar taint. Many of these non-synonymous SNPs either potentially affect the expression of the gene or alter the amino acid sequence and likely affect the activity of the gene product, but this needs to be confirmed from gene expression studies.

Another potential application of genetic markers is to use them to guide treatment protocols for boar taint. This would involve testing different treatments for boar taint in groups of pigs and comparing the response to different treatments for boar taint to the genotype of the pigs. This would allow us to identify those pigs that respond to treatment based on their genetic marker profile. Thus, we propose the use of genetic markers as biomarkers to guide strategies for controlling boar taint in pigs with specific genotypes.

#### 4.6.2. Gene Editing

Another potential genetic approach to reduce boar taint is using gene editing [101]. Gene editing differs dramatically from older transgenic technology where novel genes were introduced into animals or gene function was knocked out (KO) to produce a ‘genetically modified organism’ (GMO). The process of producing a transgenic animal required the use of a selectable marker such as antibiotic resistance to identify cells carrying the transgene. The transgenic animal also carried this gene, and this was one of the reasons for the consumer resistance to ’GMO’ products [102]. In contrast, genome editing allows the introduction of specific mutations or novel sequences targeted to a specific place in the genome without the use of a selectable marker.

The application of genome editing in livestock species [103] and specifically in pig [104] has been recently reviewed. The most efficient genome editing tool is the CRISPR/Cas9 system that originated as a defense mechanism of bacteria against foreign viral DNA. The Cas9 nuclease introduces double strand breaks in specific sites on DNA and is guided to this target site by two non-coding RNAs. The double strand breaks are then repaired in two different ways. The error-prone non-homologous end joining method introduces insertions and deletions during the repair to knock out gene function. Alternatively, introducing a DNA template to guide homologous direct repair makes single or multiple base pair changes possible. 

Gene editing involves specifically targeting and altering the genes that affect a particular trait, and this has already been done with the poll gene to produce cattle without horns, the myostatin gene for double muscling or altering disease resistance such as for African Swine Fever and PRRS [103]. Animals generated using genome editing could also be combined with artificial gene drives to increase genetic gain for favourable alleles in breeding programs in a short time [105].

There are currently two gene-editing approaches under development to control boar taint. These are based on delayed puberty by knocking out the *KISS1R* gene that is involved in the onset of puberty [106] or inactivating the *SRY* gene involved in testis development [101]. These strategies will prevent the formation of the boar taint steroid androstenone but will also reduce the production of the androgens and estrogens that are responsible to the superior growth performance of boars; this will also likely result in difficulty in reproduction of the edited animals. 

We have developed a strategy for genome editing to control boar taint that involves specifically altering the genes that are involved in the first step in the biosynthesis of 16-androstene steroids (androstadienol) while maintaining the production of the sex steroid DHEA [25]. This approach specifically targets the boar taint problem and still maintains the advantage of the male phenotype and superior growth performance of males. We have investigated several target sites in porcine CYB5A that decrease the stimulatory effect of CYB5A on the synthesis of androstadienol by CYP17A1, while maintaining the normal production of DHEA. We have also investigated residues in the steroid binding pocket of CYP17A1 and on the surface of CYP17A1 that are involved in binding to CYB5A to decrease the synthesis of androstadienol. We found that the CYP17A1 mutation D103S in combination with the CYB5A mutations N21K, L28V, N21K/L28V or R25M/N62S was most effective at reducing the production of androstadienol while maintaining the production of DHEA from pregnenolone using a cell-based in vitro system [25]. Introduction of these mutations into pigs using gene-editing techniques may lead to the production of low-boar-taint pigs that still have the superior production characteristics of entire males.

The public acceptance of the use of gene-editing technology has been studied in southern Brazil [107]. Just over half of respondents considered gene modification of male pigs acceptable, based on improved animal welfare, with those not in favor being opposed to genetic modification. Unforeseen consequences of genetic modification was a major concern of 80% of participants. So far, the EU considers organisms created using gene-editing techniques to be classified as genetically modified organisms (GMOs), so the use of gene-editing technology in the EU is limited.

## 5. Developing Custom Solutions for Boar Taint

As described above, boar taint is a multifactorial problem that is affected by a number of physiological, nutritional, environmental and genetic factors (Figure 1 and Figure 4). Furthermore, there is too much variability in these factors between breeds and individuals within a breed to implement a blanket solution for boar taint. We propose a precision livestock management approach that integrates the information on the various factors affecting boar taint to develop custom treatments based on the cause of boar taint for specific groups or individuals. 

The relative importance of different physiological processes that contribute to boar taint in individual animals needs to be determined. We propose comparing the genotypes of functional mutations of individual animals with expression of key genes and activity of key pathways affecting the levels of androstenone or skatole. This will allow the genetic profiling of functional mutations in metabolic genes involved with boar taint and/or developing proteomic or metabolic profiles associated with boar taint. These marker profiles can then be used to determine the cause of boar taint in individuals and serve as biomarkers to determine which of the solutions to boar taint will work for individual animals. 

Some examples of treatments are the use of dietary additives and changes to the gut microflora to affect production of skatole, use of binding agents to interrupt the enterohepatic recirculation of androstenone, activation of nuclear receptors using natural products to alter gene expression and affect the synthesis and/or metabolism of androstenone and skatole.

### 5.1. Control of Androstenone

Androstenone accumulation can be affected by the rate of synthesis of free and sulfated 16-androstene steroids in Leydig cells, their rate of metabolism in the liver and the rate of enterohepatic circulation from the gut back into the blood stream. For androstenone synthesis, the expression of key genes and the activity of key enzymes involved in biosynthesis of androstenone should be assessed. We also need to consider the extent of sulfoconjugation of androstenone. There may also be differences in androstenone transport in blood and subsequent deconjugation and uptake of 16-androstene steroids by adipocytes and muscle cells. For androstenone metabolism, pigs that produce high levels of androstenone should be identified, for example by measuring plasma levels of androstenone at 21 days of age. In these studies, the expression of genes and activity of enzymes involved in metabolism and clearance of androstenone in liver and the importance of enterohepatic circulation of androstenone should be assessed. A profile of functional polymorphisms in genes involved in these functions should be developed.

### 5.2. Control of Skatole

Skatole levels can be affected by the rate of synthesis of skatole in the gut, including the effect of the composition of the gut microbiome, the rate of uptake from the intestine, and the rate of degradation in the liver and subsequent excretion.

For skatole metabolism, the levels of skatole in feces should be measured to identify high producing individuals to be used for metabolic clearance studies. The microbiome profiles associated with high and low skatole production should be identified. For skatole metabolism, the expression of key liver enzymes and polymorphisms in these genes should be determined. Levels of skatole metabolites in plasma and urine can be assessed to identify rapid and slow metabolizers of skatole.

## 6. Conclusions

Boar taint is a multifactorial problem and effective and practical alternatives to surgical castration to control boar taint are needed. There are various pre-existing methods that can reduce the accumulation of androstenone or skatole in the fat; however, many studies have failed to establish the efficacy of these methods using animals that have high fat concentrations of androstenone and skatole. It is therefore not only important for future research in this area to define the boar taint phenotype of all animals, but to also ensure that animals with significant concentrations of boar taint compounds in the fat are included in these studies. The development of novel solutions to boar taint will require a more complete understanding of the integrated metabolism of boar taint compounds and the causes for the variations in boar taint development among different breeds or among individual pigs. Therefore, there is a need to integrate metabolic, physiological and genomic information to define the processes that lead to differences in boar taint among breeds and individuals within a breed. This will require differences in the synthesis or the metabolism of boar taint compounds in different animals to be identified in order to characterize the factors that make some animals more susceptible than others to developing boar taint. Measuring the activity of key enzymes and the expression of key genes in these metabolic pathways in pigs with different levels of boar taint can help to define these differences, which will allow us to design novel and effective individualized treatment strategies to control boar taint without castration.
